# When Time Is of the Essence: Multifocal Infective Endocarditis in a Young Patient

**DOI:** 10.7759/cureus.114343

**Published:** 2026-08-11

**Authors:** Lara E Budwig, Paul Njoku, Amit K.J. Mandal, Constantinos G Missouris

**Affiliations:** 1 Internal Medicine, Wexham Park Hospital, Frimley Health NHS Foundation Trust, Slough, GBR; 2 Cardiology, Wexham Park Hospital, Frimley Health NHS Foundation Trust, Slough, GBR; 3 Internal Medicine and Cardiology, Wexham Park Hospital, Frimley Health NHS Foundation Trust, Slough, GBR; 4 Cardiology, University of Nicosia Medical School, Nicosia, CYP

**Keywords:** bilateral infective endocarditis, congenital heart surgery, heart valve diseases, infective endocarditis complications, multivalvular infective endocarditis, paediatric cardiology, pulmonary valve replacement, s. mitis endocarditis, transoesophageal echocardiography

## Abstract

Infective endocarditis (IE) is uncommon in adolescents but may cause severe morbidity, especially with congenital heart disease (CHD) or delayed diagnosis.

A 17-year-old male with a presumed closed perimembranous ventricular septal defect (VSD) presented with five months of weight loss, malaise, fevers, cough and pleuritic chest pain. A new pansystolic murmur prompted transthoracic echocardiography (TTE), which was highly suggestive of mitral and pulmonary valve vegetations with evidence of severe valvular regurgitation. Blood cultures grew *Streptococcus mitis *and computed tomography (CT) demonstrated pulmonary septic emboli. Progressive multivalvular destruction required transfer to a tertiary adult CHD and cardiothoracic centre where he underwent complex cardiac surgery. He underwent mitral and tricuspid valve repair, pulmonary valve replacement, VSD closure and resection of a double-chambered right ventricle (DCRV). Postoperative complete atrioventricular (AV) block required temporary epicardial pacing, but intrinsic rhythm remained stable and AV conduction subsequently recovered.

This case highlights the impact of diagnostic delay in adolescent IE and the importance of early identification, multidisciplinary team (MDT) management and prompt intervention in this patient cohort.

## Introduction

Infective endocarditis (IE) is a life-threatening infection of the endocardial surface, associated with substantial morbidity and mortality through valvular destruction, cardiac dysfunction and systemic embolic complications [1]. Although IE is more commonly encountered in adults, it remains an important but uncommon diagnosis in children and adolescents, with an estimated annual incidence of 0.43-0.69 cases per 100,000 children [2]. Congenital heart disease (CHD), comprising structural abnormalities of the heart or great vessels that are present from birth, is the leading predisposing factor for IE in this age group [2-4]. Ventricular septal defects (VSDs) are particularly relevant because high-velocity turbulent flow across the defect can damage the adjacent endocardium, creating a nidus for bacterial adhesion during episodes of bacteraemia [4,5]. 

The presentation of paediatric IE may be insidious and non-specific, frequently resulting in diagnostic delay and advanced valvular involvement or embolic complications before definitive treatment is started. This diagnostic challenge is particularly relevant when a congenital defect is small or believed to have closed spontaneously, as clinically significant residual shunting may persist. 

We report a case of tri-valvular IE involving the pulmonary, mitral and tricuspid valves in an adolescent male with a small perimembranous VSD. The disease was complicated by pulmonary septic embolisation and required complex cardiac surgery and temporary epicardial pacing for postoperative complete atrioventricular (AV) block, followed by recovery of conduction and a favourable clinical outcome. This case highlights the importance of maintaining a high index of suspicion for IE in young patients with CHD and prolonged systemic symptoms. It also emphasises the value of early echocardiography, timely surgical intervention and coordinated multidisciplinary care in optimising acute and long-term outcomes. 

## Case presentation

A 17-year-old South Asian male with a background of mild global developmental delay and a perimembranous VSD, previously thought to have closed spontaneously, presented for routine paediatric cardiology review. He reported five months of malaise, 10 kg weight loss, intermittent fever, pleuritic chest pain and persistent cough, plus three weeks of back pain. He had received several antibiotic courses for presumed lower respiratory tract infection without sustained improvement. Clinical examination was notable for a new grade 3/6 pansystolic murmur at the left sternal edge and no signs of heart failure. Transthoracic echocardiogram (TTE) demonstrated large mobile vegetations (>10 mm) visible on the pulmonary and mitral valves with severe regurgitation, consistent with native multivalvular IE. A small perimembranous VSD with left-to-right flow was seen. He was subsequently sent to the local emergency department and admitted to the coronary care unit under the cardiology team for further management. 

Day 1 of Admission

**Figure 1:**
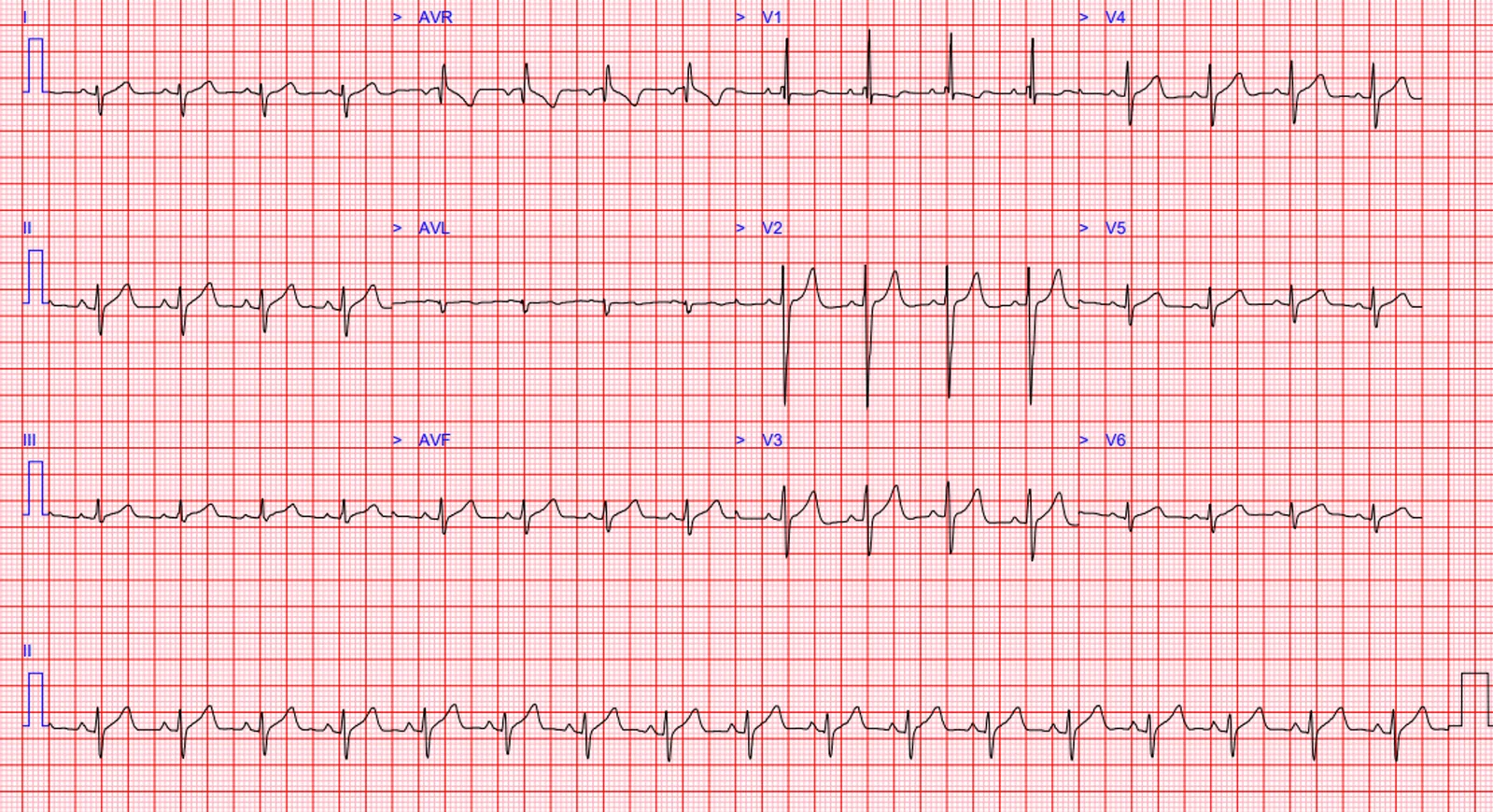
Admission 12-lead electrocardiogram (ECG) demonstrating normal sinus rhythm.

On admission, he remained febrile, with temperatures reaching 39.4°C, but was haemodynamically stable. The pansystolic murmur was unchanged, and there were no peripheral stigmata of IE or clinical features of heart failure. His admission 12-lead electrocardiogram (ECG) demonstrated sinus rhythm with a normal PR interval of 125 ms (Figure 1). Initial laboratory investigations showed a C-reactive protein (CRP) of 81 mg/L, with a normal white cell count (WCC) of 6.7 × 10⁹/L, neutrophil count of 5.1 × 10⁹/L and haemoglobin of 129 g/L (Table 1). Urine and serial blood cultures were obtained prior to antimicrobial therapy. Following microbiology discussion, empirical intravenous amoxicillin, ceftriaxone and gentamicin were commenced.

**Table 1:** Biochemistry results on admission. g/L: grams per litre; mg/L: milligrams per litre

Blood test	Result	Laboratory reference ranges
Haemoglobin (g/L)	129	130 - 180
White cell count (x 10^9^/L)	6.7	4 - 11
Neutrophils (x 10^9^/L)	5.1	2 - 7.5
C-reactive protein (mg/L)	81	0 - 5

Day 2 

**Figure 2:**
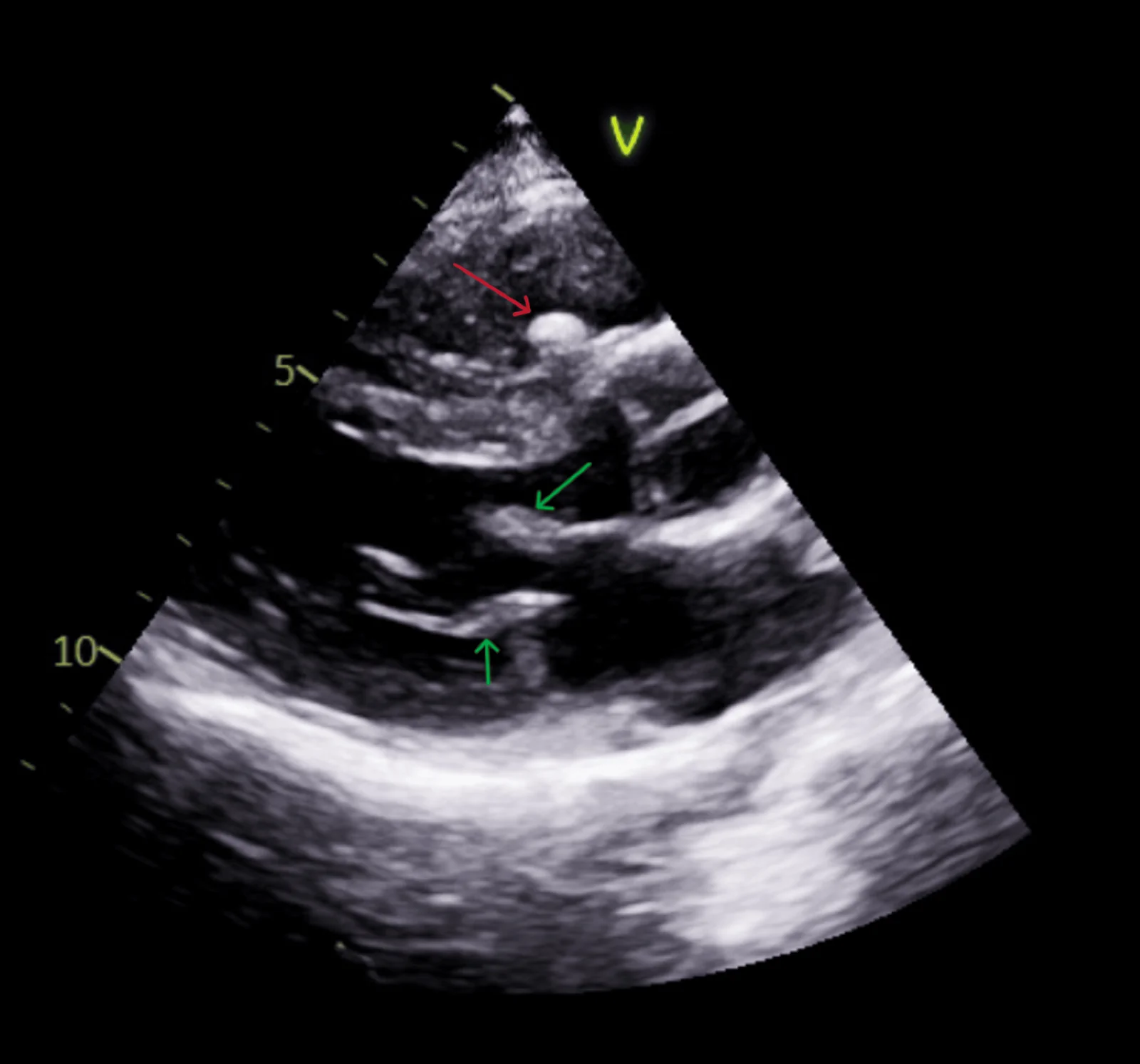
Transthoracic echocardiography demonstrating a mobile echogenic mass present on the mitral valve leaflets (green arrows) and in the right ventricular outflow tract appearing to be attached to the pulmonic valve (red arrow) (parasternal long-axis view).

**Figure 3:**
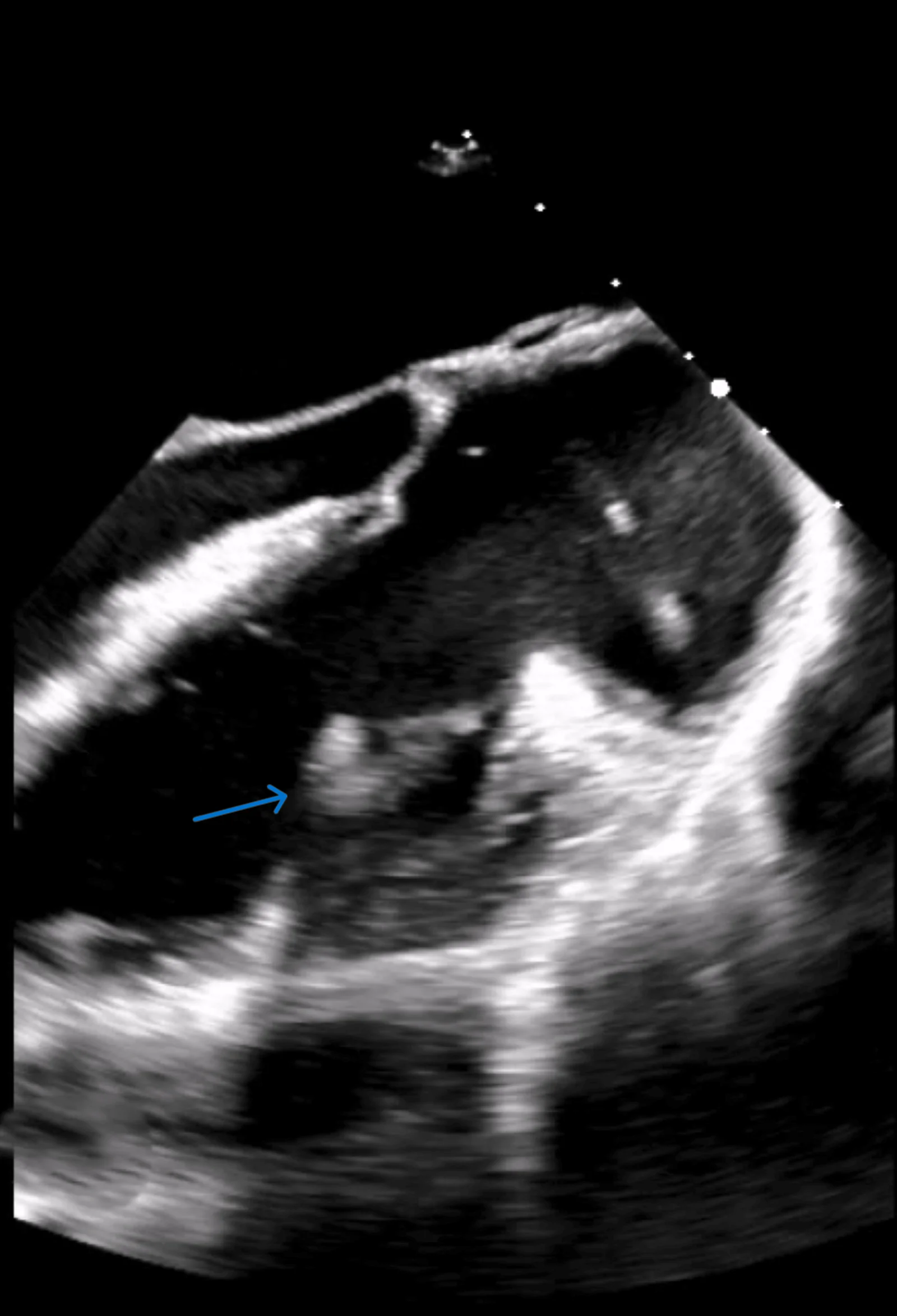
Transthoracic echocardiogram demonstrating a mobile mass consistent with a vegetation on the posterior mitral valve leaflet (blue arrow) (apical 2-chamber view).

Repeat TTE showed the presence of a mobile echogenic mass on the outflow side attached to the pulmonic valve (0.64 × 1.10 cm), with mild regurgitation. A mobile echogenic structure was also visualised attached to the posterior mitral valve (PMV) leaflet (1.02 × 0.27 cm), with moderate regurgitation (Figures 2-3) and preserved left ventricular (LV) systolic function (LV ejection fraction, 64%). Mild tricuspid regurgitation was also seen. The known perimembranous VSD could not be appreciated on this study. 

Day 3 

Urine culture was sterile, while blood cultures grew penicillin-sensitive *Streptococcus mitis*. Infectious disease advice was for six weeks of intravenous amoxicillin and gentamicin from the first negative blood culture. Inflammatory markers rose further (CRP 115 mg/L, WCC 12.0 x 10^9^/L, neutrophils 8.7 x 10^9^/L) (Table 2). 

**Table 2:** Biochemistry results on day 3 of admission. g/L: grams per litre; mg/L: milligrams per litre

Blood test	Result	Laboratory reference ranges
Haemoglobin (g/L)	116	130 - 180
White cell count (x 10^9^/L)	12.0	4 - 11
Neutrophils (x 10^9^/L)	8.7	2 - 7.5
C-reactive protein (mg/L)	115	0 - 5

Day 4 

**Figure 4:**
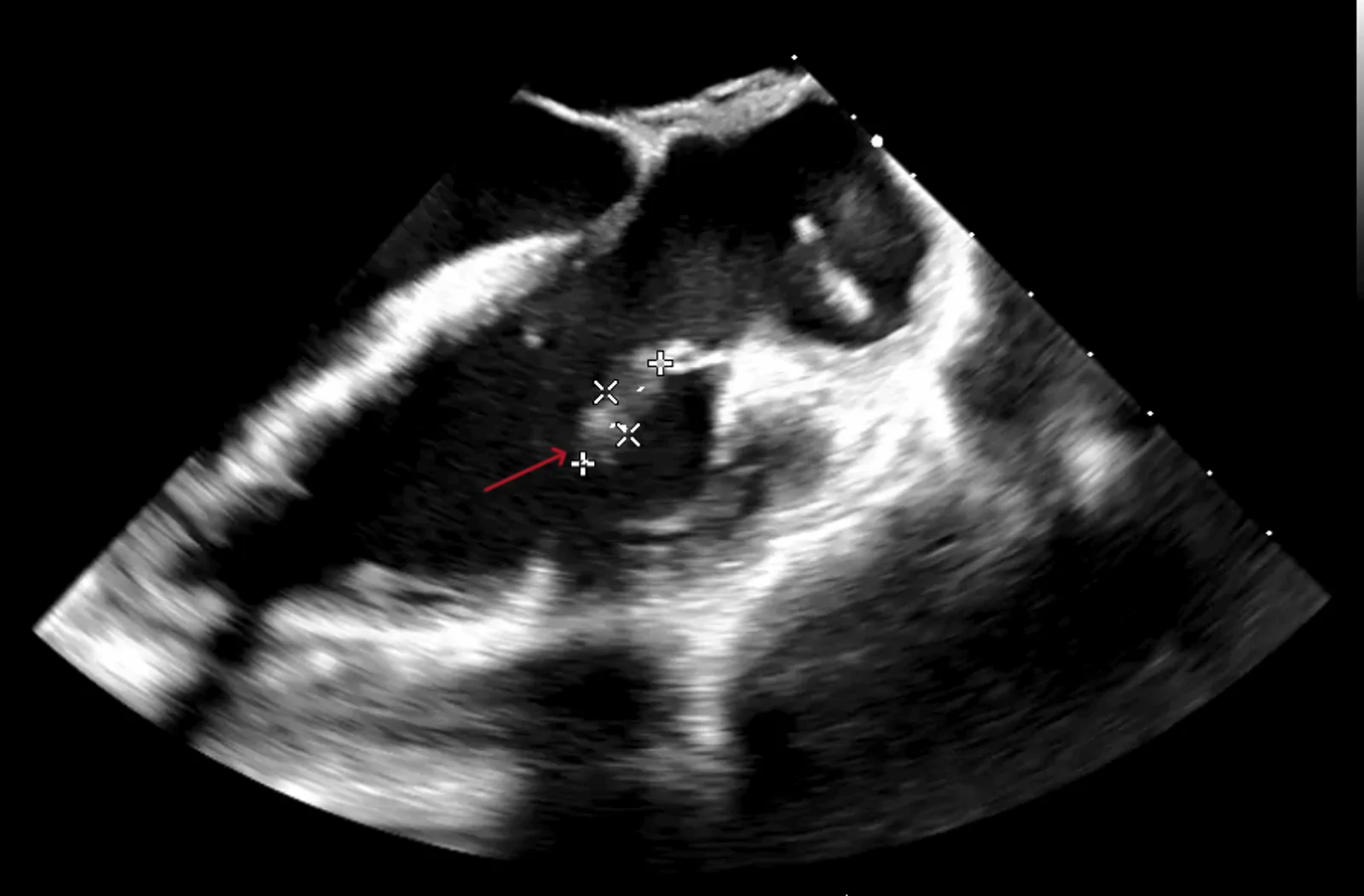
Transoesophageal echocardiogram (TOE) demonstrating a mobile echogenic mass on the posterior mitral valve leaflet in keeping with a vegetation measuring 0.8 cm x 0.28 cm (red arrow) (apical 2-chamber view).

**Figure 5:**
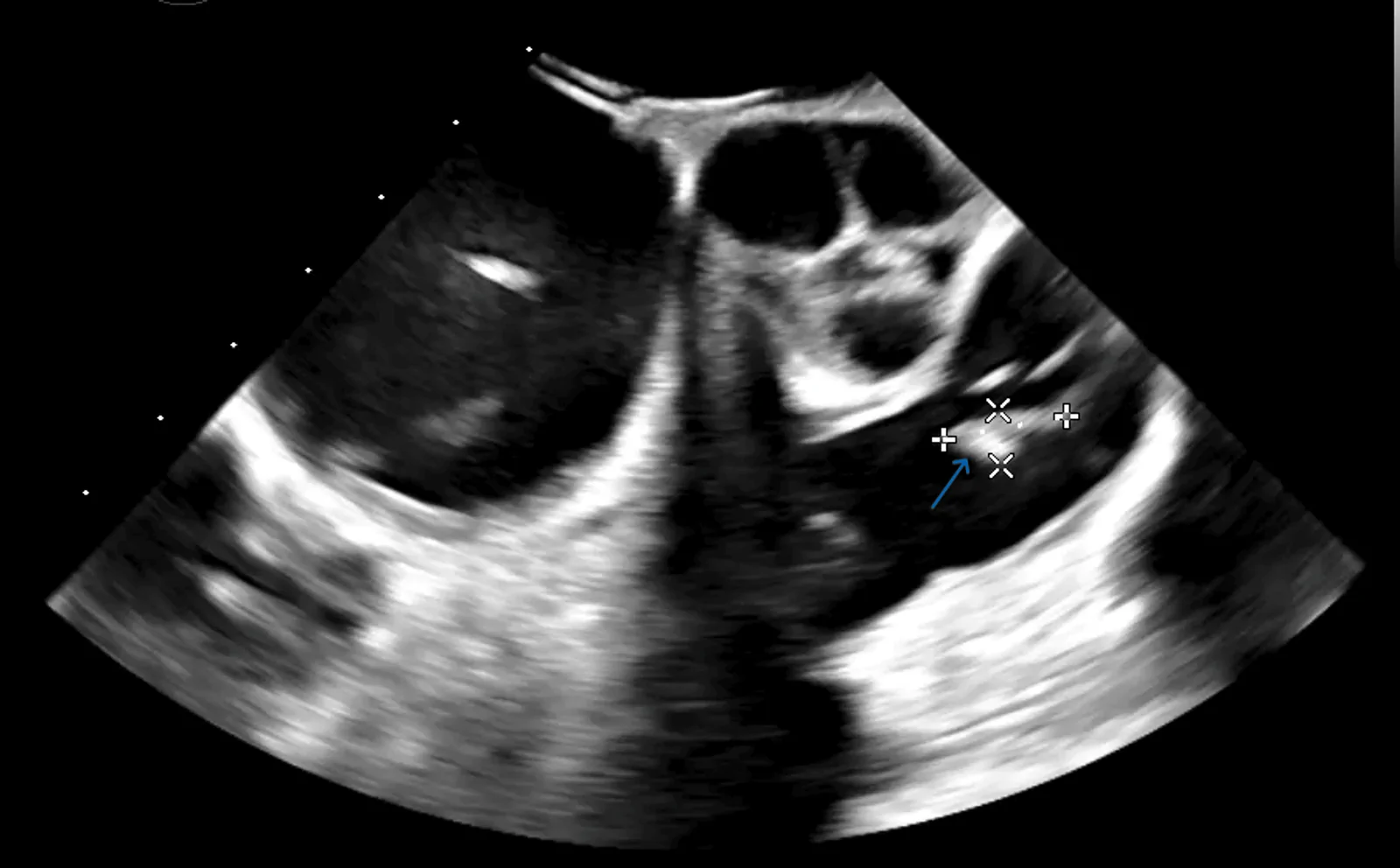
Transoesophageal echocardiogram (TOE) image demonstrating a mobile echogenic mass attached to the pulmonic valve (1.2 cm × 0.5 cm) (blue arrow) (mid-oesophageal right ventricular inflow-outflow view).

**Figure 6:**
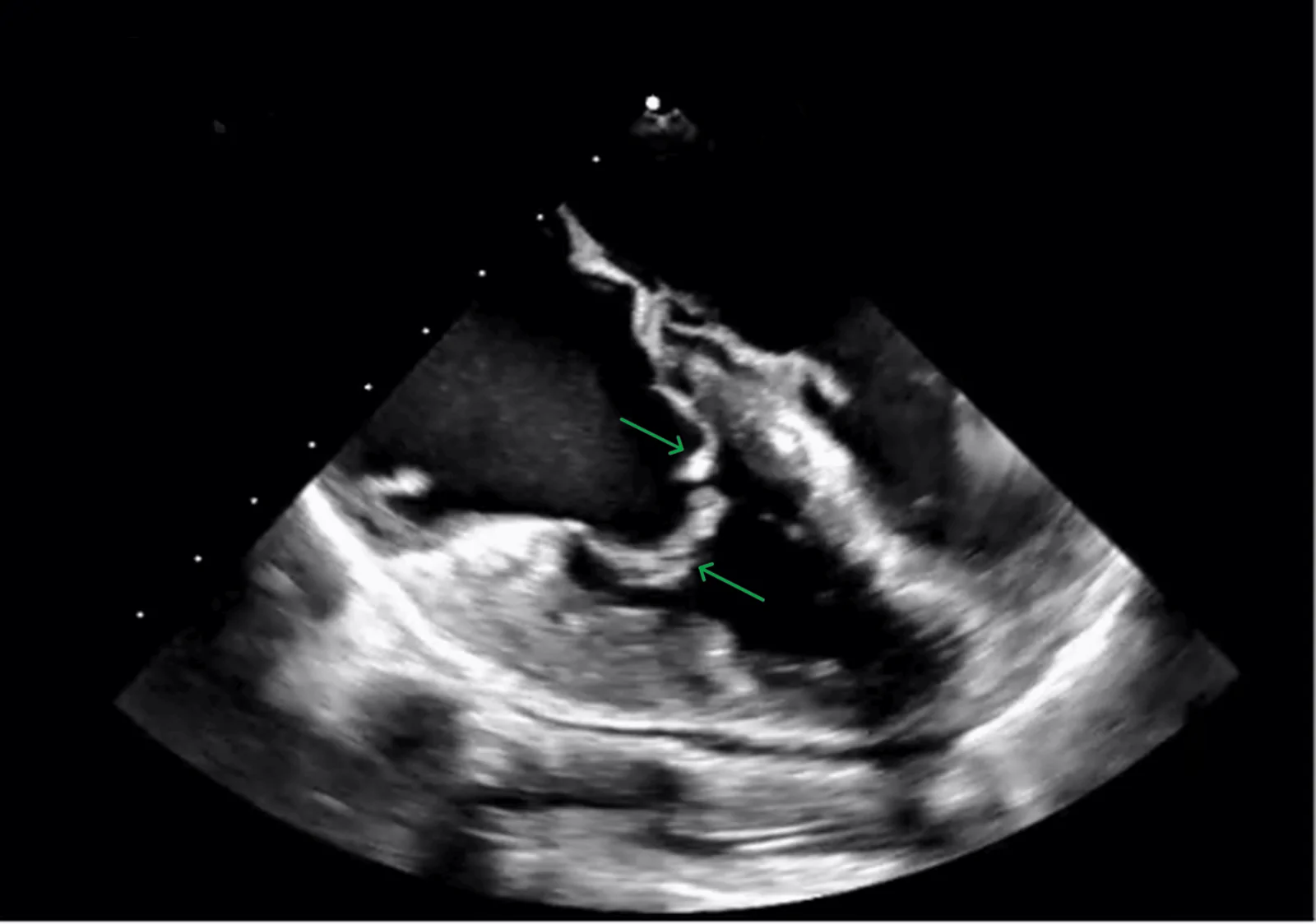
Transoesophageal echocardiogram (TOE) image showing thickening of the tricuspid valve leaflets (green arrows) (mid-oesophageal four chamber view).

Transoesophageal echocardiography (TOE) confirmed florid vegetations of the PMV leaflet (0.8 x 0.28 cm) (Figure 4) and pulmonic valve (1.2 x 0.5 cm) (Figure 5), both with severe regurgitation and possible perforation of the mitral valve. The aortic valve and root were unaffected. The tricuspid valve base was thickened (Figure 6) with mild-to-moderate regurgitation.

Day 5 

**Figure 7:**
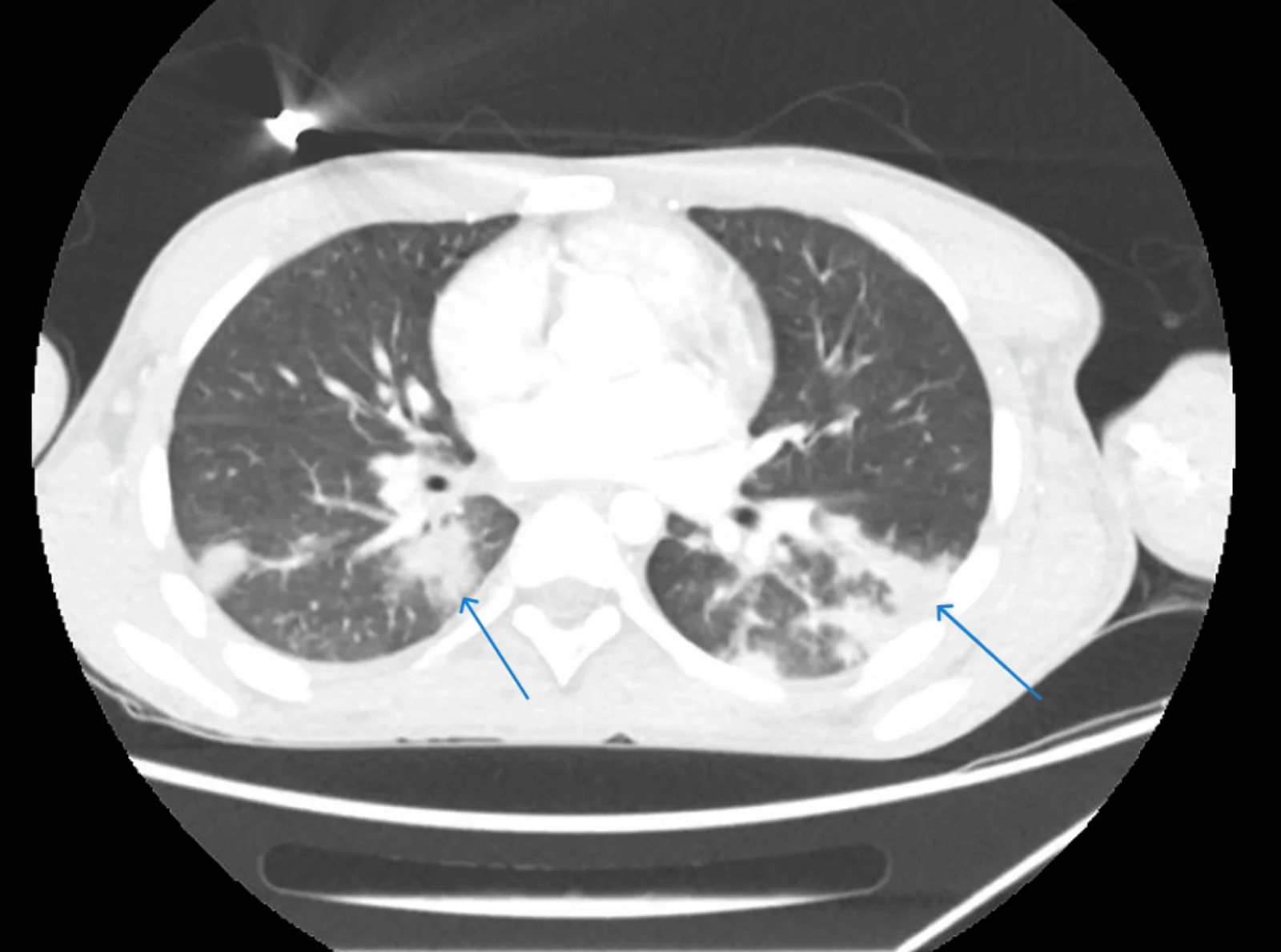
Computed tomography (CT) imaging of the lung parenchyma demonstrating bilateral patchy consolidation involving the right and left lower lobes (blue arrows) suggestive of septic embolisation.

**Figure 8:**
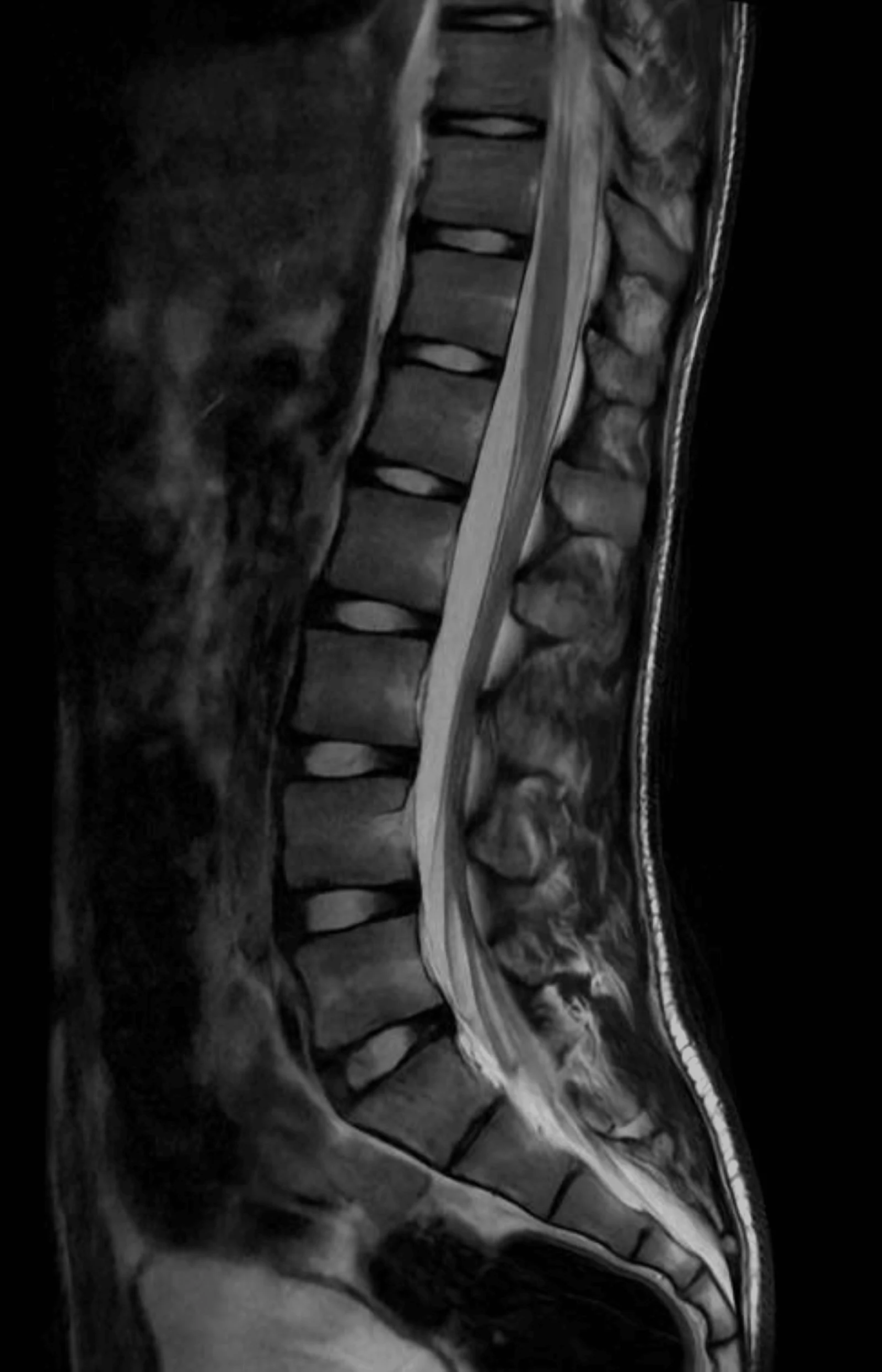
Magnetic resonance imaging (MRI) of the lumbosacral spine demonstrating normal spinal alignment and bone marrow signal with no evidence of discitis or vertebral osteomyelitis.

Computed tomography (CT) of the thorax, abdomen and pelvis showed bilateral patchy pulmonary consolidation consistent with septic emboli, but was otherwise unremarkable (Figure 7). Magnetic resonance imaging (MRI) of the spine excluded discitis and vertebral osteomyelitis (Figure 8). CRP increased to 221 mg/L (Table 3). 

**Table 3:** Biochemistry results on day 5 of admission. g/L: grams per litre; mg/L: milligrams per litre

Blood test	Result	Laboratory reference ranges
Haemoglobin (g/L)	120	130 - 180
White cell count (x 10^9^/L)	11.5	4 - 11
Neutrophils (x 10^9^/L)	8.0	2 - 7.5
C-reactive protein (mg/L)	221	0 - 5

Day 6 

Given the severe valvular disease, worsening inflammatory markers and concern for further embolisation, the case was discussed with the regional cardiothoracic and adult congenital heart disease (ACHD) multidisciplinary team (MDT), who recommended urgent transfer to a tertiary centre for early surgery to reduce embolic risk and prevent further myocardial damage.

Day 7 - Arrival at Tertiary Cardiothoracic Centre

On arrival at the tertiary centre, he remained clinically unwell and had recently been febrile, with a productive cough following influenza, bilateral pulmonary consolidation and a persistently elevated CRP level of 224 mg/L (Table 4). These findings raised concern for a possible superimposed bacterial respiratory infection. While the referring hospital’s microbiological results were being verified, repeat blood cultures and respiratory samples were obtained and antimicrobial therapy was temporarily broadened empirically to intravenous piperacillin-tazobactam and vancomycin pending further microbiological results and urgent surgery. 

**Table 4:** Biochemistry results on day 7 of admission. g/L: grams per litre; mg/L: milligrams per litre

Blood test	Result	Laboratory reference ranges
Haemoglobin (g/L)	120	130 - 180
White cell count (x 10^9^/L)	13.1	4 - 11
Neutrophils (x 10^9^/L)	9.5	2 - 7.5
C-reactive protein (mg/L)	224	0 - 5

He also underwent maxillofacial review given the *S. mitis* bacteraemia and possible odontogenic source. He reported a six-month history of dental pain and had not had a recent dental review. No dental abscess or obvious cavitation was identified, although tenderness involving the right lower canine and first molar was noted. Postoperative dental follow-up was advised. Repeat TTE and TOE confirmed the previously described multivalvular vegetations and regurgitant lesions. A double-chambered right ventricle (DCRV) was visualised on the TTE with moderate mid-cavity obstruction with a peak gradient of 47 mmHg. Following review by the anaesthetic and cardiothoracic surgical teams, he was optimised and prepared for surgical intervention. 

Day 9 (Day of Surgery)

Intraoperative findings demonstrated destructive vegetations involving the pulmonary and mitral valves. The anterior and septal leaflets of the tricuspid valve were thickened, with appearances consistent with vegetation. A tight DCRV with a septal vegetation and mid-cavity obstruction was also identified, along with a small VSD. All resected tissue specimens were sent for microbiological analysis, and cultures from all operative specimens showed no microbial growth.

He underwent successful mitral and tricuspid valve repair, prosthetic pulmonary valve homograft replacement, VSD closure and DCRV resection. Postoperative TOE showed a well-seated pulmonary valve, satisfactory mitral and tricuspid valve repair with trivial regurgitation and no residual septal shunt. He was transferred to the Intensive Care Unit (ICU) and developed postoperative complete AV block requiring temporary epicardial pacing. 

Day 11 (Two Days After Surgery)

Postoperative TTE showed good mitral, tricuspid and pulmonary valve function, no pericardial effusion and no residual septal shunt. A peripherally inserted central catheter (PICC) line was inserted, and antibiotics were simplified to once-daily intravenous ceftriaxone. 

Day 13 (Four Days After Surgery)

By postoperative day 4, he remained in complete AV block but had a stable intrinsic escape rhythm with haemodynamic stability and no ongoing pacing requirement. He was therefore deemed not pacing-dependent and the temporary pacing wires were removed. 

Day 15 (Six Days After Surgery)

**Figure 9:**
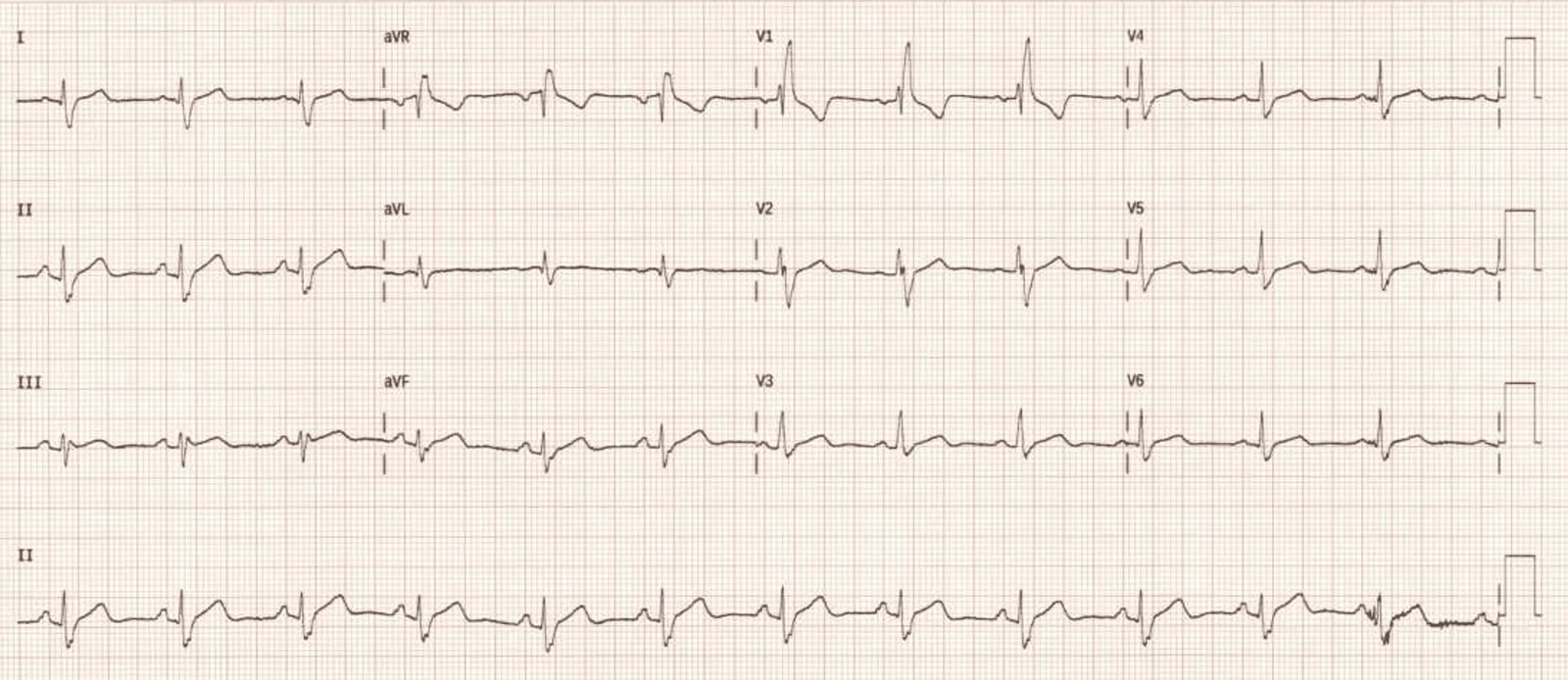
Post-operative 12-lead electrocardiogram (ECG) demonstrating sinus rhythm with right bundle branch block (RBBB).

After good postoperative recovery and rehabilitation, he was stepped down to the general cardiology ward for cardiac rehabilitation and physiotherapy. Dental assessment showed adequate dentition with no clear source for the bacteraemia. AV conduction subsequently recovered and follow-up ECG demonstrated sinus rhythm with right bundle branch block (RBBB) (Figure 9). Repeat TTE was unchanged. 

Day 19 (Day of Discharge - 10 Days After Surgery)

He remained afebrile and asymptomatic, with a well-healed sternotomy wound, improving inflammatory markers and sterile repeat blood cultures. His CRP had fallen to 10 mg/L. He was discharged with a plan for daily ambulatory intravenous ceftriaxone to complete a six-week course of antimicrobial therapy, calculated from the date of the first negative blood culture. He was also scheduled for weekly ACHD follow-up for four weeks, all of which were uneventful.

Six-Month Follow-Up

He remained clinically well with no evidence of recurrent IE. Surveillance TTE demonstrated satisfactory mitral and tricuspid valve repair and a well-functioning prosthetic pulmonary valve. His 12-lead ECG showed sinus rhythm with RBBB and no recurrence of high-grade AV block. 

He was advised to maintain meticulous oral hygiene and attend regular dental review. In view of his history of IE and prosthetic valve replacement, antibiotic prophylaxis was recommended for invasive dental procedures involving manipulation of the gingival tissue or perforation of the oral mucosa, in accordance with specialist endocarditis guidance as per the European Society of Cardiology.

## Discussion

IE is uncommon in children and adolescents, with an estimated annual incidence of 0.43-0.69 cases per 100,000 children [2]. CHD remains the leading predisposing factor in this age group [2-4]. VSDs increase the risk through turbulent intracardiac blood flow, which may cause endothelial disruption and create a nidus for bacterial adhesion during episodes of bacteraemia. Importantly, even small or apparently functionally closed defects may remain clinically relevant if residual turbulence or minor endocardial injury persists [4,5]. 

Viridans group streptococci, including *S. mitis*, are well-recognised causes of IE and are frequently associated with oral disease, dental manipulation or poor dentition [6,7]. Although no definitive dental source was identified in this case, the patient reported several months of dental pain and had not undergone recent dental review. This highlights the importance of oral hygiene, routine dental surveillance and prompt treatment of dental infection in patients with CHD. Current National Institute for Health and Care Excellence (NICE) guidance does not recommend routine antibiotic prophylaxis for all dental procedures, but it recognises that patients with structural cardiac abnormalities, previous IE, acquired valvular disease or prosthetic valves are at increased risk of IE, reinforcing the importance of individualised risk assessment and preventative dental care [8-10].

The diagnosis of IE in adolescents can be challenging because the clinical presentation is often insidious and non-specific [2,3]. In this case, the patient’s prolonged history of fever, weight loss, malaise, cough and pleuritic chest pain was initially treated as recurrent respiratory infection. IE was only suspected after the detection of a new murmur during routine cardiology review. This emphasises the need for a high index of suspicion in young patients with CHD who present with persistent systemic symptoms, even in the absence of overt heart failure or classical peripheral stigmata of IE.

Multimodality imaging plays a central role in the diagnosis, anatomical characterisation and risk stratification of IE [11]. TTE identified valvular vegetations and significant regurgitation, while TOE provided further definition of vegetation size, valve involvement and structural complications. CT subsequently demonstrated pulmonary septic emboli, a recognised complication of right-sided or multivalvular IE and an important marker of disease burden [9,12]. In complex CHD-associated IE, early integration of echocardiography, cross-sectional imaging and microbiological findings is essential to guide timely multidisciplinary decision-making.

Targeted antibiotic therapy remains the cornerstone of IE management. In patients with established surgical indications, timely intervention may limit progressive valvular destruction and reduce embolic risk [9]. A systematic review and meta-analysis of 21 studies found that early surgery, defined as intervention within 20 days, was associated with lower all-cause mortality than delayed surgery or medical management [13]. 

This case underscores the value of coordinated multidisciplinary care in IE, with close collaboration between specialist congenital cardiology and cardiothoracic surgical teams being central to timely intervention and favourable clinical outcomes [14]. The patient required complex intervention, including mitral and tricuspid valve repair, pulmonary valve replacement, VSD closure and resection of a DCRV. Postoperative complete heart block requiring temporary pacing likely reflected the anatomical proximity of the VSD and right ventricular outflow tract surgery to the conduction system. Subsequent recovery of conduction and good functional outcome further support the value of coordinated perioperative management in specialist centres. 

## Conclusions

Although rare in adolescents, IE presents significant diagnostic and management challenges, particularly in patients with underlying CHD. As survival into adolescence and adulthood improves for patients with congenital cardiac lesions, clinicians must remain alert to late complications, including IE, even when defects are thought to have closed or become clinically insignificant. This case of multivalvular *S. mitis* endocarditis highlights the importance of long-term specialist follow-up, early echocardiography in the presence of persistent systemic symptoms, prompt microbiological diagnosis, timely surgical referral and preventative strategies, including regular dental care and antibiotic prophylaxis, in high-risk patients.

## References

[1] Cahill TJ, Prendergast BD (2016). Infective endocarditis. Lancet.

[2] Vicent L, Luna R, Martínez-Sellés M (2022). Pediatric infective endocarditis: a literature review. J Clin Med.

[3] Baltimore RS, Gewitz M, Baddour LM (2015). Infective endocarditis in childhood: 2015 update: a scientific statement from the American Heart Association. Circulation.

[4] Leonard EJ, Kuebler BE, Zenni MM (2017). A review of infective endocarditis associated with congenital heart disease. Consultant.

[5] Knirsch W, Nadal D (2011). Infective endocarditis in congenital heart disease. Eur J Pediatr.

[6] Yallowitz AW, Decker LC (2023). Infectious endocarditis. StatPearls [Internet].

[7] Bumm CV, Folwaczny M (2021). Infective endocarditis and oral health: a narrative review. Cardiovasc Diagn Ther.

[8] Habib G, Lancellotti P, Antunes MJ (2015). 2015 ESC guidelines for the management of infective endocarditis: the task force for the management of infective endocarditis of the European Society of Cardiology (ESC). Endorsed by: European Association for Cardio-Thoracic Surgery (EACTS), the European Association of Nuclear Medicine (EANM). Eur Heart J.

[9] Baddour LM, Wilson WR, Bayer AS (2015). Infective endocarditis in adults: diagnosis, antimicrobial therapy, and management of complications: a scientific statement for healthcare professionals from the American Heart Association. Circulation.

[10] (2026). National Institute for Health and Care Excellence. Prophylaxis against infective endocarditis: antimicrobial prophylaxis against infective endocarditis in adults and children undergoing interventional procedures. NICE Clinical Guideline 64. https://www.nice.org.uk/guidance/cg64.

[11] Moscatelli S, Leo I, Bianco F (2023). The role of multimodality imaging in patients with congenital heart disease and infective endocarditis. Diagnostics (Basel).

[12] Cabezon G, Pulido P, López Díaz J (2024). Embolic events in infective endocarditis: a comprehensive review. Rev Cardiovasc Med.

[13] Anantha Narayanan M, Mahfood Haddad T, Kalil AC (2016). Early versus late surgical intervention or medical management for infective endocarditis: a systematic review and meta-analysis. Heart.

[14] Roy AS, Hagh-Doust H, Abdul Azim A (2023). Multidisciplinary teams for the management of infective endocarditis: a systematic review and meta-analysis. Open Forum Infect Dis.

